# Health-Related Quality of Life in Metastatic, Hormone-Sensitive Prostate Cancer: ENZAMET (ANZUP 1304), an International, Randomized Phase III Trial Led by ANZUP

**DOI:** 10.1200/JCO.21.00941

**Published:** 2021-12-20

**Authors:** Martin R. Stockler, Andrew J. Martin, Ian D. Davis, Haryana M. Dhillon, Stephen D. Begbie, Kim N. Chi, Simon Chowdhury, Xanthi Coskinas, Mark Frydenberg, Wendy E. Hague, Lisa G. Horvath, Anthony M. Joshua, Nicola J. Lawrence, Gavin M. Marx, John McCaffrey, Ray McDermott, Margaret McJannett, Scott A. North, Francis Parnis, Wendy R. Parulekar, David W. Pook, M. Neil Reaume, Shahneen Sandhu, Alvin Tan, Thean Hsiang Tan, Alastair Thomson, Francisco Vera-Badillo, Scott G. Williams, Diana G. Winter, Sonia Yip, Alison Y. Zhang, Robert R. Zielinski, Christopher J. Sweeney

**Affiliations:** ^1^ NHMRC Clinical Trials Centre, University of Sydney, Sydney, New South Wales, Australia; ^2^ Monash University, Melbourne, Victoria, Australia; ^3^ CEMPED: The University of Sydney Centre for Medical Psychology and Evidence-Based Decision-Making, Sydney, NSW, Australia; ^4^ Port Macquarie Base Hospital, Port Macquarie, New South Wales, Australia; ^5^ BC Cancer Agency Vancouver Centre, Vancouver, BC, Canada; ^6^ Guy's and St Thomas' NHS Foundation Trust, London, United Kingdom; ^7^ Chris O'Brien Lifehouse, Sydney, New South Wales, Australia; ^8^ Kinghorn Cancer Centre, St Vincent's Hospital, Sydney, New South Wales, Australia; ^9^ Auckland District Health Board, Auckland, New Zealand; ^10^ Sydney Adventist Hospital, Sydney, NSW, Australia; ^11^ Cancer Trials Ireland, Dublin, Ireland; ^12^ St Vincent's University Hospital, Dublin, Ireland; ^13^ ANZUP Cancer Trials Groups, Sydney, NSW, Australia; ^14^ Cross Cancer Institute, Edmonton, Alberta, Canada; ^15^ Adelaide Cancer Centre, Adelaide, South Australia, Australia; ^16^ Canadian Cancer Trials Group, Kingston, Ontario, Canada; ^17^ Monash Health, Melbourne, Victoria, Australia; ^18^ University of Ottawa, Ottawa, Ontario, Canada; ^19^ Peter MacCallum Cancer Centre, Melbourne, Victoria, Australia; ^20^ Waikato District Health Board, Hamilton, New Zealand; ^21^ Royal Adelaide Hospital, Adelaide, South Australia, Australia; ^22^ Royal Cornwall Hospital, Cornwall, United Kingdom; ^23^ Kingston Health Sciences Centre, Kingston, Ontario, Canada; ^24^ Orange Health Service, Orange, New South Wales, Australia; ^25^ Dana-Farber Cancer Institute, Boston, MA

## Abstract

**PURPOSE:**

We previously reported that enzalutamide improved overall survival when added to standard of care in metastatic, hormone-sensitive prostate cancer. Here, we report its effects on aspects of health-related quality of life (HRQL).

**METHODS:**

HRQL was assessed with the European Organisation for Research and Treatment of Cancer core quality-of-life questionnaire and QLM-PR25 at weeks 0, 4, 12, and then every 12 weeks until progression. Scores from week 4 to 156 were analyzed with repeated measures modeling to calculate group means and differences. Deterioration-free survival was from random assignment until the earliest of death, clinical progression, discontinuation of study treatment, or a worsening of 10 points or more from baseline in fatigue, physical function, cognitive function, or overall health and quality of life (OHQL). HRQL scores range from 0 (lowest possible) to 100 (highest possible).

**RESULTS:**

HRQL was assessed in 1,042 of 1,125 participants (93%). Differences in means favored control over enzalutamide for fatigue (5.2, 95% CI, 3.6 to 6.9; *P* < .001), cognitive function (4.0, 95% CI, 2.5 to 5.5; *P* < .001), and physical function (2.6, 95% CI, 1.3 to 3.9; *P* < .001), but not OHQL (1.2, 95% CI, −0.2 to 2.7; *P* = .1). Deterioration-free survival rates at 3 years, and log-rank *P* values comparing the whole distributions, favored enzalutamide over control for OHQL (31% *v* 17%; *P* < .0001), cognitive function (31% *v* 20%; *P* = .001), and physical function (31% *v* 22%; *P* < .001), but not fatigue (24% *v* 18%; *P* = .16). The effects of enzalutamide on HRQL were independent of baseline characteristics.

**CONCLUSION:**

Enzalutamide was associated with worsening of self-reported fatigue, cognitive function, and physical function, but not OHQL. Enzalutamide was associated with improved deterioration-free survival for OHQL, physical function, and cognitive function because delays in disease progression outweighed early deteriorations in these aspects of HRQL.

## INTRODUCTION

Enzalutamide is a potent, oral, nonsteroidal, androgen receptor antagonist that increased overall survival in men with castration-resistant prostate cancer.^1,2^ ENZAMET (NCT02446405) is an ANZUP-led, international, randomized, phase III, cooperative group trial of enzalutamide versus an active control (physician's choice of bicalutamide, nilutamide, or flutamide), used together with testosterone suppression, in recently diagnosed, metastatic, hormone-sensitive prostate cancer (HSPC). In 2019, we reported that treatment with early enzalutamide improved overall survival in metastatic HSPC within 3 years of follow-up.^3^

**CONTEXT**

**Key Objective**
Enzalutamide prolonged overall survival and delayed progression when added to testosterone suppression for hormone-sensitive, metastatic prostate cancer in the ENZAMET trial. Determination of the effects of open-label enzalutamide on aspects of health-related quality of life (HRQL) was a prespecified secondary objective of ENZAMET.
**Knowledge Generated**
Enzalutamide was associated with worse mean self-ratings of fatigue, physical function, and cognitive function, but not overall health and quality of life. However, clinically significant declines attributable to enzalutamide occurred in a minority of participants. The adverse effects of enzalutamide on HRQL were additive to those of early docetaxel. These effects were most evident early in the treatment course. Enzalutamide was associated with net benefits in deterioration-free survival at 3 years despite these effects on HRQL.
**Relevance**
Effects on HRQL are important when considering the addition of a potent inhibitor of androgen signaling to testosterone suppression in men with hormone-sensitive, metastatic prostate cancer.

Here, we report on aspects of health-related quality of life (HRQL), a key secondary outcome in ENZAMET. Our aim was to determine the effects of enzalutamide versus active control on HRQL until clinical progression. These analyses include comparisons of HRQL scores from baseline until clinical progression and of deterioration-free survival, a novel measure of net benefit accounting for both disease control and worsening of HRQL.

## METHODS

Participants had prostate cancer with metastases evident on conventional imaging (computed tomography and/or radioisotope bone scanning with technetium) and were randomly assigned, in a 1:1 ratio, to open-label treatment with either enzalutamide 160 mg daily or an active control (physician's choice of bicalutamide, nilutamide, or flutamide), together with testosterone suppression (luteinizing hormone–releasing hormone analog or orchidectomy). Random assignment was central and used minimization with a random element to stratify for volume of disease (high volume defined as the presence of visceral metastases and/or at least four bone lesions with at least one beyond the vertebral column and pelvis),^4^ planned use of early chemotherapy with docetaxel, planned use of bone antiresorptive therapy, a comorbidity score of 2 or more according to the Adult Comorbidity Evaluation 27^5^, and study site. Three months after ENZAMET had opened and accrued 88 men, the Protocol (online only) was amended to allow the use of early chemotherapy with docetaxel after presentation of results from CHAARTED.^4^ Planned use of early chemotherapy with docetaxel was declared before random assignment and comprised six cycles at a starting dose of 75 mg/m^2^ of body surface area. The Protocol had ethics approval at all participating institutions, and all participants provided signed, written, informed consent.

### HRQL Assessments

Assessments of HRQL were scheduled to occur at baseline, weeks 4 and 12, and then every 12 weeks until clinical progression. Clinical progression was defined by conventional imaging, development of symptoms attributable to cancer progression, or initiation of another treatment for prostate cancer.

Validated instruments were used to assess pertinent aspects of HRQL. The European Organisation for Research and Treatment of Cancer (EORTC) core quality-of-life questionnaire comprises 30 items aggregated into multi-item scales for functioning (physical, role, cognitive, emotional, and social), symptoms (fatigue, pain, and nausea and vomiting), and overall health and quality of life (OHQL).^6^ The remaining single items assess additional symptoms commonly reported by people with cancer including dyspnea, appetite loss, sleep disturbance, constipation, and diarrhea. The European Organisation for Research and Treatment of Cancer disease-specific module for prostate cancer (QLM-PR25) includes multi-item scales assessing symptoms (urinary, bowel, and related to hormonal treatment), sexual activity, sexual function, and use of incontinence aids.^7^ Items about incontinence aids were completed by too few participants to report. Scores are scaled to range from 0 (worst possible) to 100 (best possible) for functioning and OHQL and from 0 (none at all) to 100 (worst possible) for symptoms. Assessments of utility elicited with the EQ-5D-5L will be reported separately as part of a health economic evaluation.^8^

### Statistical Considerations

Primary and secondary analyses were specified a priori in a statistical analysis plan written before their conduct and reflected two ways of viewing effects on HRQL. The first was to summarize and compare self-ratings HRQL from random assignment until clinical progression. The second was to describe and compare times to first deterioration defined either by a clinically important worsening in a domain of HRQL or by clinical progression.

For HRQL until progression, scores over time were summarized by the treatment group and analyzed using a repeated measures model (RMM) to calculate predicted group means, differences, 95% CIs, and *P* values on the basis of Wald tests. The RMM was specified within the general linear-mixed model framework, used a compound symmetry covariance matrix, and included covariates for randomly assigned treatment, baseline assessment, postbaseline assessment time point, and an interaction term for treatment-by-time point, all as fixed effects. The interaction term was removed to calculate the difference between groups across all postbaseline time points. Reference-based sensitivity analyses with multiple imputations were used to assess the effects of assuming HRQL data were missing at random.

Deterioration-free survival was formulated a priori as an end point reflecting net benefit during the interval from random assignment until clinical progression.^9^ It was defined as the time from random assignment until the earliest of the following events: a 10-point or greater deterioration from baseline in the pertinent aspect of HRQL (without a subsequent 10-point or greater improvement compared with baseline), clinical progression, treatment discontinuation, or death from any cause. Participants who experienced none of these events had their observations censored at the date they were last known not to have clinically progressed.

The two end points for deterioration-free survival formulated a priori used different indicators of a deterioration in HRQL on the basis of the core quality of life questionnaire: One used the scale for physical function and the other used the scale for OHQL. The criterion of a 10-point reduction to signify a clinically important deterioration was specified a priori on the basis of evidence-based guidelines.^10^ Given the known effects of enzalutamide on fatigue and self-reported cognitive function, we also performed post hoc analyses of deterioration-free survival focused on these aspects.

Distributions of deterioration-free survival by the randomly assigned treatment group were compared with the log-rank test and summarized by percentages alive and deterioration-free at 3 years calculated with the Kaplan-Meier method. Hazard ratios and 95% CIs were calculated with Cox proportional hazards models.

Post hoc, subgroup analyses were used to examine if the effects of enzalutamide on HRQL were modified by baseline characteristics, particularly planned use of early chemotherapy with docetaxel. This was done by augmenting the previously described models by adding a main effect term for the characteristic and an interaction term for the effect of enzalutamide by characteristic. Evidence of effect modification is indicated by a low *P* value for the interaction term.

Analyses included all participants who completed an assessment at baseline and at least one additional postbaseline time point. All CIs and *P* values are two-sided, without correction for multiplicity, and should be interpreted conservatively as evidence against the null hypothesis.

## RESULTS

HRQL data were extracted from the study database on December 31, 2019, with a cutoff date of February 28, 2019, to match our first reported analysis of overall survival.^4^

HRQL was reported by 1,042 of 1,125 (93%) participants. Baseline characteristics were similar among those randomly assigned enzalutamide versus control (Table 1) and among those who did and did not report HRQL (Data Supplement, online only). Scores for aspects of HRQL at baseline were similar among those assigned enzalutamide versus control (Table 2). Characteristics associated with worse OHQL at baseline included impaired ECOG performance status, metastatic disease at initial diagnosis, and high-volume disease, but not planned use of early chemotherapy with docetaxel or age 70 years and older (Table 3).

**TABLE 1.**
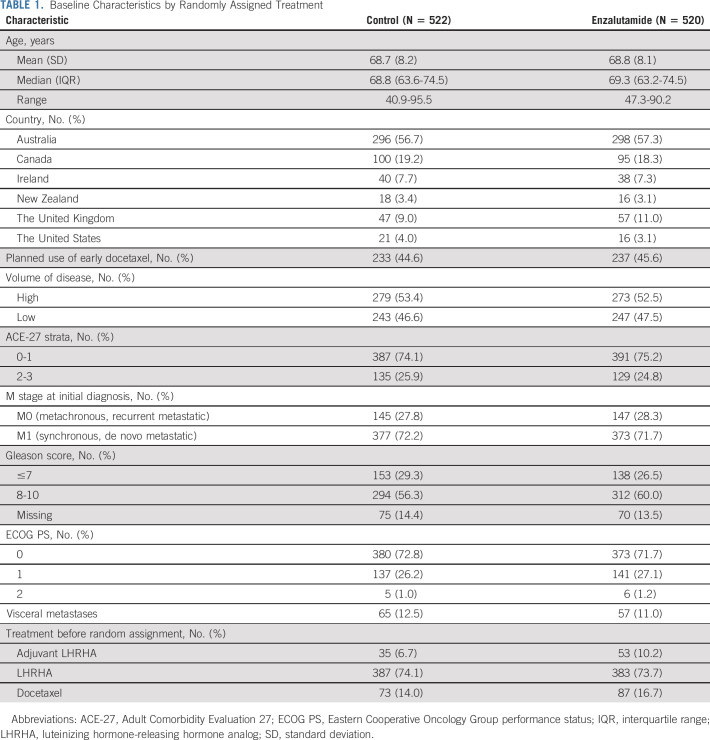
Baseline Characteristics by Randomly Assigned Treatment

**TABLE 2.**
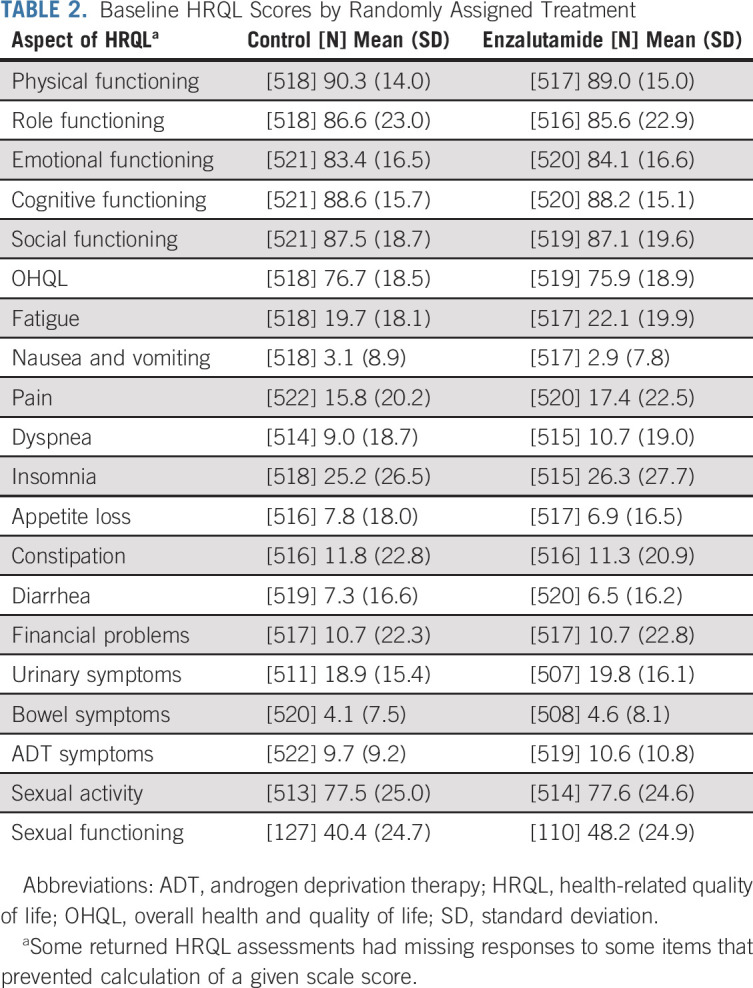
Baseline HRQL Scores by Randomly Assigned Treatment

**TABLE 3.**
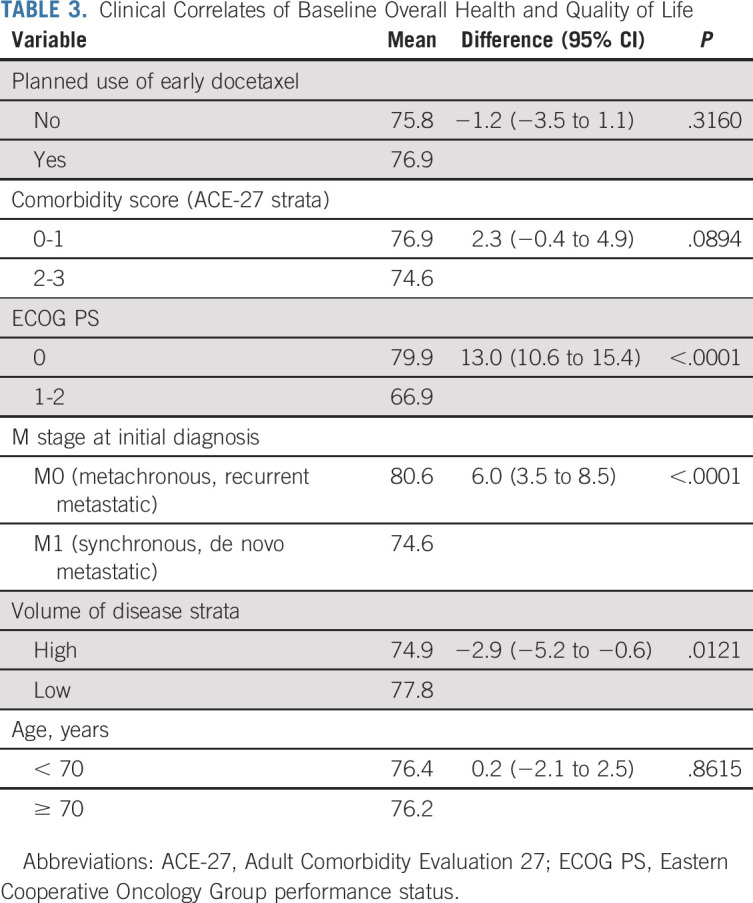
Clinical Correlates of Baseline Overall Health and Quality of Life

The median follow-up time was 34 months. The proportions of HRQL assessments expected that were received ranged from 93% at 4 weeks to 86% at 156 weeks (Data Supplement).

### HRQL Over Time

**FIG 1.**
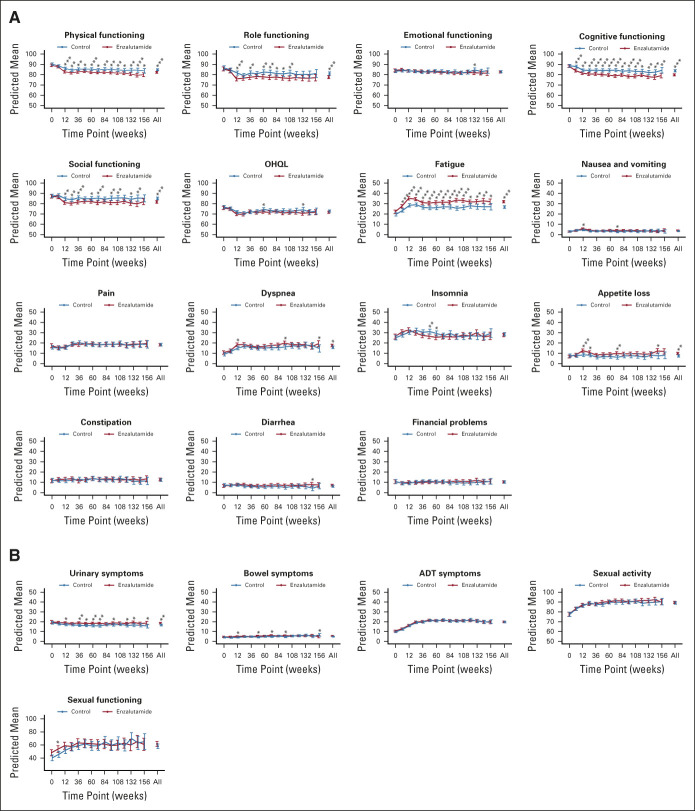
Health-related quality of life scores over time by randomly assigned treatment for (A) the EORTC core quality-of-life questionnaire and (B) EORTC QLM-PR25. *P < .05, **P < .01, ***P < .001. ADT, androgen deprivation therapy; EORTC, European Organisation for Research and Treatment of Cancer; EORTC QLM-PR25, European Organisation for Research and Treatment of Cancer disease-specific module for prostate cancer; OHQL, overall health and quality of life.

Mean scores for aspects of HRQL by the randomly assigned treatment group are shown for each assessment point, and overall, in Figure 1 and the Data Supplement. OHQL differed little between men randomly assigned to enzalutamide versus control (overall means 72 *v* 73, difference 1.2, 95% CI, 0.2 to 2.7; *P* = .10). Scores at 12 weeks deteriorated to a greater extent in the enzalutamide group than controls (means 70 *v* 72, difference 1.9, 95% CI, −0.1 to 4.0; *P* = .07) and thereafter remained relatively stable.

Differences in overall means for functional domains favored control over enzalutamide for physical functioning (2.6, 95% CI, 1.3 to 3.9; *P* < .001), role functioning (3.6, 95% CI, 1.5 to 5.7; *P* = .001), social functioning (3.3, 95% CI, 1.5 to 5.1; *P* < .001), and cognitive functioning (4.0, 95% CI, 2.5 to 5.5; *P* < .001), but not for emotional functioning (*P* = .8). The trajectories for these functional domains were similar to those for OHQL.

Differences in overall means for selected symptoms favored control over enzalutamide for fatigue (5.2, 95% CI, 3.6 to 6.9; *P* < .001), appetite loss (2.5, 95% CI, 1.0 to 4.0; *P* = .001), urinary symptoms (1.9, 95% CI, 0.6 to 3.1; *P* = .003), and dyspnea (1.8, 95% CI, 0.0 to 3.5; *P* = .05), but not pain, nausea and vomiting, insomnia, constipation, diarrhea, hormonal symptoms, and sexual activity or sexual dysfunction (all *P* > .08).

The observed differences between the randomly assigned treatment groups in HRQL scores over time, and their upper 95% CI, were less than the predefined clinically important difference of 10 points for all aspects of HRQL.

Sensitivity analyses yielded similar results and conclusions. Boxplots of actual HRQL scores over time and of predicted scores from the RMM can be found in the Data Supplement. Sensitivity analyses assuming unstructured covariances can be found in the Data Supplement.

**FIG 2.**
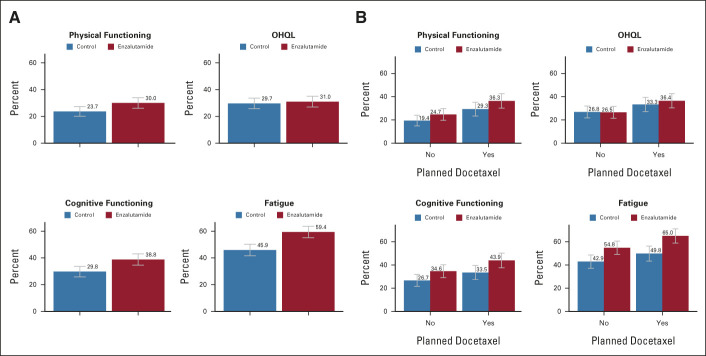
Early deteriorations in health-related quality of life (A) by randomly assigned treatment for the whole group and (B) by physician-assigned plan for early chemotherapy with docetaxel. OHQL, overall health and quality of life.

Early deteriorations in key aspects of HRQL were more evident with enzalutamide than control for physical function, fatigue, and cognitive function, but not OHQL (Fig 1). To explore these early effects, we evaluated the frequency of a 10-point or greater deterioration from baseline during the first 12 weeks as a specific, patient-reported indicator of the adverse effects of treatment (Fig 2A). This confirmed that early deteriorations in HRQL were more frequent among men assigned enzalutamide than control for fatigue (difference 13%, 95% CI, 7 to 20), cognitive function (9%, 95% CI, 3 to 15), and physical function (6%, 95% CI, 1 to 12), but not OHQL (1.3%, 95% CI, −4 to 7). These analyses also showed that early deteriorations were common in the control group, untreated with enzalutamide, ranging from 24% of participants for physical functioning to 46% of participants for fatigue. These frequencies included participants treated with early docetaxel, so we also evaluated the frequencies of early deteriorations in subgroups classified by physician-assigned plan for early chemotherapy with docetaxel (Fig 2B). These analyses confirmed that early deteriorations were also common in control group members untreated with early chemotherapy, ranging from 19% of participants for physical functioning to 43% for fatigue, and that the modest detrimental effects associated with enzalutamide were additive and independent to those of concurrent treatment with early docetaxel. Similar effects were seen on the frequencies of 10-point or greater improvements from baseline to 12 weeks (Data Supplement).

### Deterioration-Free Survival

**FIG 3.**
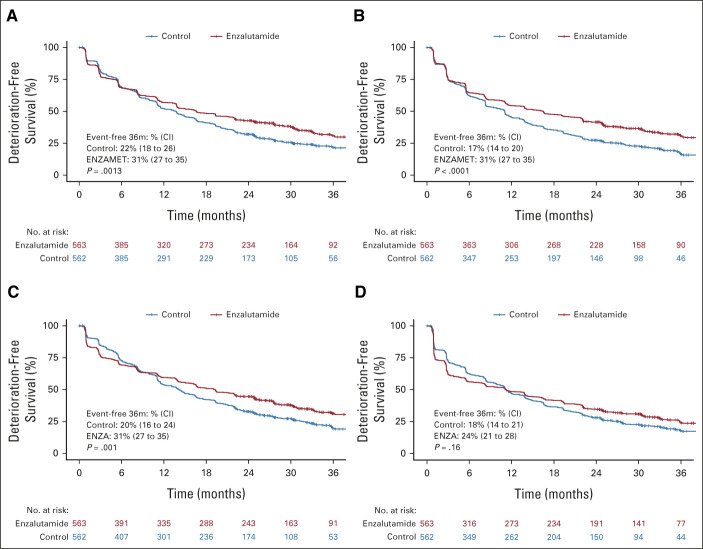
Kaplan-Meier curves for deterioration-free survival by randomly assigned treatment: (A) physical functioning, (B) OHQL, (C) cognitive functioning, and (D) fatigue. OHQL, overall health and quality of life.

Kaplan-Meier curves for deterioration-free survival by the randomly assigned treatment group are shown in Figure 3. Drops in the curves over the first 3 months, mainly attributable to early deteriorations in HRQL because of treatment, were greater with enzalutamide than control for physical function, fatigue, and cognitive function, but not OHQL. However, benefits in disease control attributable to enzalutamide resulted in crossing of the curves beyond 6 months such that improved deterioration-free survival rates at 3 years, and log-rank *P* values comparing the whole distributions, favored enzalutamide over control for physical functioning (31% *v* 22%; *P* = .0013), cognitive functioning (31% *v* 20%; *P* < .001), and OHQL (31% *v* 17%; *P* < .001), but not fatigue (24% *v* 18%; *P* = .16). Cumulative incidence functions separating the components of deterioration-free survival can be found in the Data Supplement.

### Early Chemotherapy With Docetaxel and Other Subgroup Effects

Subgroup analyses on the basis of interaction tests for prespecified baseline characteristics recorded before random assignment were used to determine if the effects of enzalutamide were modified by these characteristics. These analyses indicated that planned early chemotherapy with docetaxel, assigned by physicians, did not modify the effect of randomly assigned enzalutamide on HRQL scores until progression (all *P* > .14, Data Supplement), deterioration-free survival (all *P* > .06, Data Supplement), or early deteriorations in HRQL (all *P* > .5, Data Supplement). These analyses indicate that the effects of enzalutamide and planned early docetaxel on HRQL were independent, incremental, and approximately additive.

## DISCUSSION

Treatment with enzalutamide was associated with worse self-ratings of fatigue, physical function, and cognitive function, but not OHQL. Differences between the randomly assigned treatment groups, and their upper 95% confidence limits, were below our prespecified threshold for a minimum clinically important difference of 10 points (on a scale from 0 to 100). These effects were apparent within 3 months of starting study treatment and remained relatively constant thereafter. Men randomly assigned to enzalutamide were more likely to have early deteriorations in fatigue, physical function, and cognitive function, regardless of early chemotherapy with docetaxel. Despite these early impairments in aspects of HRQL, delays in clinical progression because of treatment with enzalutamide led to net benefits in deterioration-free survival. Mean scores for aspects of HRQL at baseline among all trial participants reflected a low burden of cancer-related symptoms and good quality of life on aggregate, similar to reference norms from a population-based sample of Australian men.^11^ However, men with high-volume disease at baseline, or with metastatic disease at initial diagnosis, reported worse OHQL than their counterparts.

Our findings add to accumulating evidence about potent inhibitors of androgen signaling in HSPC. Enzalutamide had little effect on OHQL in ENZAMET, similar to findings from the ARCHES trial of enzalutamide,^12^ the TITAN trial of apalutamide,^13^ and the LATITUDE trial of abiraterone.^14^ However, unlike ARCHES, TITAN, and LATITUDE, we report that modest detrimental effects of enzalutamide on fatigue, physical function, and self-rated cognitive function, that occurred early, were of a clinically important magnitude in a minority of patients and did not worsen over time. The detrimental effects of enzalutamide on aspects of HRQL were additive and independent of concurrent treatment with early docetaxel. ENZAMET differs from ARCHES, TITAN, and LATITUDE in two important ways. First, ENZAMET was an open-label trial, rendering it susceptible to over-reporting of adverse effects documented in the participant information. Second, ENZAMET was the only trial that tested a novel antiandrogen given concurrently with docetaxel; ARCHES, TITAN, and LATITUDE required completion of early chemotherapy before random assignment.

The main strengths of this study are its randomized design, large sample size, high completion of HRQL assessments, active treatment in the control group, and concurrent use of enzalutamide with docetaxel in 48% of participants. The study had high power to detect clinically important differences between treatment groups and clinically important modifications of the treatment effect in major subgroups. These strengths support our conclusion that the impairments in HRQL associated with enzalutamide were generally modest and of similar magnitude, regardless of planned early chemotherapy with docetaxel.

The main limitations of these analyses are that HRQL was assessed only until progression and for a maximum of 3 years. HRQL beyond progression is affected by subsequent treatments that were left to treating physicians. The follow-up time of 3 years matches our first interim analysis of overall survival. Follow-up is ongoing to determine the effects of enzalutamide beyond 3 years.

The addition of early enzalutamide to standard of care, including testosterone suppression with or without early docetaxel, improved overall survival during 3 years of follow-up with modest impairments in fatigue, physical function, and self-rated cognitive function, but not OHQL, in men with recently diagnosed, metastatic, HSPC. These impairments did not worsen over time, and better disease control improved deterioration-free survival rates at 3 years because the modest detrimental effects on aspects of HRQL were outweighed by subsequent delays in disease progression. Longer follow-up will reveal the effects of enzalutamide beyond 3 years.
